# TriNymAuth: Triple Pseudonym Authentication Scheme for VANETs Based on Cuckoo Filter and Paillier Homomorphic Encryption

**DOI:** 10.3390/s23031164

**Published:** 2023-01-19

**Authors:** Luyuan Zhuang, Nan Guo, Yufan Chen

**Affiliations:** Computer Science & Engineering College, Northeastern University, Shenyang 110167, China

**Keywords:** VANETs, Paillier homomorphic encryption, cuckoo filter, identity authentication, privacy protection

## Abstract

In VANETs, owing to the openness of wireless communication, it is necessary to change pseudonyms frequently to realize the unlinkability of vehicle identity. Moreover, identity authentication is needed, which is usually completed by digital certificates or a trusted third party. The storage and the communication overhead are high. This paper proposes a triple pseudonym authentication scheme for VANETs based on the Cuckoo Filter and Paillier homomorphic encryption (called TriNymAuth). TriNymAuth applies Paillier homomorphic encryption, a Cuckoo Filter combining filter-level and bucket-level, and a triple pseudonym (homomorphic pseudonym, local pseudonym, and virtual pseudonym) authentication to the vehicle identity authentication scheme. It reduces the dependence on a trusted third party and ensures the privacy and security of vehicle identity while improving authentication efficiency. Experimental results show that the insert overhead of the Cuckoo Filter is about 10 μs, and the query overhead reaches the ns level. Furthermore, TriNymAuth has significant cost advantages, with an OBU enrollment cost of only 0.884 ms. When the data rate in VANETs dr≤ 180 kbps, TriNymAuth has the smallest total transmission delay cost and is suitable for shopping malls and other places with dense traffic.

## 1. Introduction

In intelligent transportation systems, Vehicular Ad Hoc Networks (VANETs) can realize real-time communication between vehicles and infrastructure. VANETs play an active role in improving traffic safety, reducing environmental pollution, alleviating traffic congestion, and providing convenient transportation. Sensitive information such as vehicle position, trajectory, and speed, will be generated in the communication process of VANETs. Generally, it is not recommended to encrypt this sensitive information to protect vehicle privacy because vehicle communication in VANETs usually needs this information.

Vehicles usually use pseudonyms instead of their true identities to realize anonymous communication. It is necessary to achieve message integrity and authenticate the identity of the communication entity in the process of message transmission. Authentication ensures that only legal and valid entities have access to sensitive information transmitted in VANETs. Furthermore, the vehicle’s location privacy is associated with the driver’s personal information because the trajectory of the vehicle usually has something to do with the driver’s personal information, which, if leaked, may endanger the driver’s life and property. Therefore, user privacy protection is very important. To achieve the timely accountability of malicious vehicles, it is necessary to realize conditional privacy protection, which ensures that only the Trusted Authority (TA) can obtain the vehicle’s real identity in a timely and effective manner and hold the malicious vehicle accountable.

At present, some anonymous authentication schemes based on the Bloom Filter (BF), which can reduce message transmission overhead and improve authentication efficiency, are proposed. The BF is a data structure with high space utilization, but the BF does not support dynamic addition and deletion of entries. In 2014, Bin et al. [1] proposed a new data structure to improve the BF, called the Cuckoo Filter (CF). The CF is a data structure for approximate set membership queries. It improves the BF in the following three aspects: (1) it supports deleting items dynamically, (2) better search performance, and (3) Better storage performance for applications that require a low false positive rate.

### 1.1. Motivation and Contributions

This paper proposes a triple pseudonym authentication scheme for VANETs based on the Cuckoo Filter and Paillier homomorphic encryption (called TriNymAuth). The goal is to solve the problems of certificate storage and certificate management in traditional authentication schemes and solve the problem that other anonymous authentication schemes rely too much on RoadSide Unit (RSU) or TA for identity authentication. Based on the efficient query and storage advantages of the CF, TriNymAuth reduces storage and computation overhead. TriNymAuth uses homomorphic pseudonym (HomoNym), local pseudonym (LocNym), and virtual pseudonym (VirNym) to realize the triple authentication of vehicle identity: (1) HomoNym solves the pseudonym self-updating problem of the OnBoard Unit (OBU), and the Homomorphic Pseudonym Provider (HPP) does not need to preload HomoNyms for vehicles, which saves the storage space of the OBU. The HPP does not need to update the vehicle’s HomoNyms online, which reduces the computational overhead of the HPP. (2) In V2V authentication, LocNym is used for vehicle identity authentication inside VANETs, which effectively prevents the HPP from associating vehicle identities inside VANETs. (3) VirNym prevents the RSU from associating LocNyms with OBU. As can be seen, triple pseudonym authentication ensures the security and privacy of vehicle identity while implementing identity authentication and reducing communication overhead between the vehicle and the RSU or the vehicle and the HPP during V2V authentication. The following presents the main contributions of TriNymAuth.

1. The idea of using triple pseudonyms is one of the main contributions of this paper.
  - HomoNym realizes the correspondence between the vehicle’s real identity ID and multiple HomoNyms, such as OBU${}_{A}$’s $\left(ID_{A},HomoNym_{Ai}\right)$. HomoNyms is updated synchronously by the HPP and OBU.
  - LocNym implements the correspondence between HomoNym and LocNym, such as OBU${}_{A}$’s $\left(HomoNym_{A},LocNym_{A}\right)$. LocNym is generated by the OBU and registered with the RSU.
  - VirNym implements the correspondence between LocNym and multiple VirNyms, such as OBU${}_{A}$’s $\left(LocNym_{A},VirNym_{Ai}\right)$, and the OBU periodically updates a set of virtual pseudonyms to be used during V2V communication. Each virtual pseudonym becomes invalid when it is used up.
  The triple pseudonyms are updated synchronously. There are two update opportunities for triple pseudonyms: (1) update in accordance with the suggested updated cycle in 5GAA [2], and (2) update when OBU drives across RSU regions.
2. The update of the vehicle’s HomoNyms does not depend on the preloading or online update of TA, which reduces the storage and communication overhead.
3. A two-stage HomoNym enrollment protocol is based on the CF. Fresh vehicles joining VANETs must apply to the RSU for HomoNym enrollment, the RSU forwards the enrollment message to the HPP, and the HPP verifies the validity of the vehicle’s HomoNym using an efficient CF query service. The vehicle does not need to sign with the root certificate private key, and the HPP does not need to verify the signature of the vehicle, which reduces the computational overhead. Inside the VANETs, vehicles use LocNyms and local private keys that are regularly updated for identity authentication and do not rely on the HPP, which improves the authentication efficiency. This separation of internal and external identity authentication in VANETs achieves identity privacy protection, unlinkability of HomoNyms, non-repudiation, and message integrity.
4. The HomoNym revocation protocol is based on Paillier homomorphic encryption and the CF. In the aspect of identity tracing (revocation), because all the HomoNyms generated in the life cycle of the vehicle are stored in the HPP, the HPP can quickly obtain the malicious vehicle’s true identity based on Paillier homomorphic decryption by using the additive property of homomorphic encryption in the abnormal situation, which realizes the traceability of vehicle identity and reduces the storage overhead. Different from other traditional authentication schemes that distribute Certificate Revocation Lists (CRL) for vehicles, TriNymAuth maintains the CRL in the CF to store revoked HomoNyms and verifies the validity of the HomoNyms by querying the CRL. This reduces the overhead of CRL distribution management.
5. The V2V authentication mechanism is based on VirNyms’ exchange and usage. A series of VirNyms are generated and exchanged between vehicles for subsequent communication. The vehicle uses the Elliptic Curve Digital Signature Algorithm (ECDSA) [3] to sign VirNyms to realize identity authentication, which not only reduces the dependence on the RSU and the HPP but also solves the key escrow problem, which is the most common problem in identity-based authentication schemes. It improves the efficiency of authentication while ensuring the privacy protection of vehicle identity, non-repudiation, and message integrity. In addition, TriNymAuth also satisfies the security and privacy requirements of unlinkability, traceability, and avoiding impersonation attacks in VANETs.

### 1.2. Paper Organization

TriNymAuth is organized as follows: (1) Section 2 is the related works section, which introduces common authentication schemes in VANETs and explains the advantages of filter-based anonymous authentication schemes and the recent advancements in this field. (2) Section 3 is the preliminaries section, which introduces the system model and related technologies involved in the scheme. (3) Section 4 is the pseudonym management scheme based on Paillier homomorphic encryption and the CF, which introduces the specific protocols involved in each stage according to the pseudonym life cycle order. (4) Section 5 is the security and privacy analysis, which introduces the security and privacy satisfied by TriNymAuth and compares it with related works. (5) Section 6 is the performance analysis, which introduces the performance advantages of the CF itself and, on this basis, gives a comparative analysis of the enrollment cost, computational cost, communication cost, and total transmission delay of the scheme.

## 2. Related Works

This section introduces common authentication schemes in VANETs and divides them into identity-based authentication schemes, BF-based authentication schemes, and CF-based authentication schemes. The specific classification is as follows:

### 2.1. Identity-Based Authentication Scheme

A lot of identity-based authentication schemes in VANETs have been proposed in recent years. In 2008, an identity-based batch verification (IBV) scheme [4] was proposed, which realized conditional privacy protection. In the IBV scheme, the RSU could verify multiple signatures simultaneously, which reduced the verification time. Moreover, in the IBV scheme, certificates were not required, which significantly reduced the transmission overhead. In 2011, Chim et al. [5] proposed a Secure and Privacy Enhancing Communications Scheme (SPECS). SPECS found that there were some limitations in the IBV scheme [4]. Firstly, the IBV scheme relied heavily on the Tamper Proof Device (TPD), which preloaded all keys within the system. Once compromised, the entire system was compromised. Secondly, the IBV scheme did not meet the privacy requirements, and the real identity of the vehicle could be traced by anyone. Thirdly, the IBV scheme could not resist an impersonation attack and an anti-traceability attack. Finally, in IBV scheme’s batch verification, if there was something wrong in one of the signatures, the whole batch would be discarded, which reduced the efficiency of signature verification.

In 2020, AL-SHAREEDA et al. [6] proposed a VANET-Based Privacy-Preserving Communication Scheme (VPPCS). Based on signatures and verification, VPPCS realized identity authentication and ensured data privacy. However, when the pseudonym set expired, the vehicle needed to delete the old pseudonym set and then request to obtain a new pseudonym set, which increased the computational overhead of the system and the storage overhead of the vehicle.

### 2.2. BF-Based Authentication Scheme

SPECS [5] not only addressed the limitations of the IBV scheme [4], but also improved space utilization, reduced the storage overhead, and addressed the storage overhead problem in VPPCS [6]. SPECS [5] proposed a new identity-based authentication scheme using the BF and a binary search algorithm that used two shared secrets to meet the privacy requirements. In 2013, Horng et al. [7] proposed batch verification for secure pseudonymous authentication (b-SPECS+). The b-SPECS+ pointed out that SPECS was also not resistant to impersonation attacks. Therefore, b-SPECS+ improved the message signing phase in SPECS, and the improved scheme could meet the security and privacy requirements.

In order to ensure VANETs’ security, it is essential to revoke the access rights of malicious nodes with a history of misconduct [8]. Certificates in the CRL refer to public key certificates. A pseudonym is actually a short-term public key. The CRL is not only a public key certificate revocation list but also a short-term public key (pseudonym) revocation list. The CRL is a common way to verify whether the certificate is revoked.

Based on the high space utilization of the BF, some schemes using the BF to store the CRL were proposed [9,10,11,12], which reduced the size of the CRL. In 2017, Zhong et al. [9] proposed a Conditional Privacy-Preserving Authentication (CPPA) scheme using the BF to reduce the size of the CRL. This scheme did not use bilinear pairing and reduced the computational overhead. However, if an RSU is compromised, the vehicles’ real identities in the range of the RSU will be obtained by the adversary. Moreover, the BF needed to be updated whenever a notification message was generated because the BF did not support dynamic increase, so the time cost was large.

### 2.3. CF-Based Authentication Scheme

In 2017, Cui et al. [13] proposed a CF-based privacy-preserving authentication scheme (SPACF). Based on the dynamic update and deletion characteristics of CF, SPACF could alleviate the problem that updating the BF takes a long time. In SPACF’s batch verification phase, the CF and binary search methods were adopted to achieve a high batch verification success rate. However, since SPACF [13] used identity-based signatures, there was a key escrow problem.

Zhang et al. [14] proposed to directly apply the CF to CRL generation and used the Certificate Expiration List (CEL) to assist the dynamic deletion of expired certificates. CEL provided more free space for storing other valid certificate fingerprints and reduced the probability of a hash collision.

In 2019, Alazzawi et al. [15] proposed a CPPA scheme for VANETs based on pseudonym roots to obtain pseudonyms. This scheme did not use bilinear pairing, had low computational overhead, did not use CRL, and reduced storage and communication overhead. However, the RSU was required to broadcast the notification messages in the CF periodically and act as the intermediate node between the vehicle and TA when the vehicles authenticated each other. The vehicle’s identity authentication relied on TA, which was difficult to deploy in areas where RSUs were not deployed or were sparsely deployed.

In 2020, a CF-based privacy-preserving authentication scheme for V2V and V2I communication was proposed in the literature [16]. The scheme used a binary search algorithm to establish positive and negative filter pools for integer key verification and improved verification efficiency through batch verification.

In this paper, TriNymAuth uses Paillier homomorphic encryption to generate and self-update the vehicle’s HomoNym, which solves the high computational overhead and reduces the overhead of storing HomoNym in OBU. The HPP uses the CF to store HomoNyms and CRL, which effectively uses the space of the HPP and avoids the overhead of distributing CRL and the overhead of managing CRL on OBU. In the two-stage HomoNym enrollment phase, the vehicle uses the HomoNym and LocNym to apply for HomoNym enrollment from the RSU. The HPP queries the HomoNym based on the CF to achieve efficient verification of HomoNym. In this phase, signatures and verification are not needed, which reduces the computational overhead. In V2V authentication of VirNyms’ exchange and usage, VirNyms based on ECDSA algorithm signatures are used to achieve identity authentication between vehicles, which avoids impersonation attacks. Moreover, TriNymAuth satisfies VANETs’ security and privacy requirements, such as unlinkability and traceability.

## 3. Preliminaries

This section describes the system model of TriNymAuth, the Cuckoo Filter for storing HomoNyms and the CRL, the Paillier encryption algorithm for homomorphic pseudonym generation and update, and the ECDSA algorithm for identity authentication during V2V communication.

### 3.1. System Model

**Figure 1:**
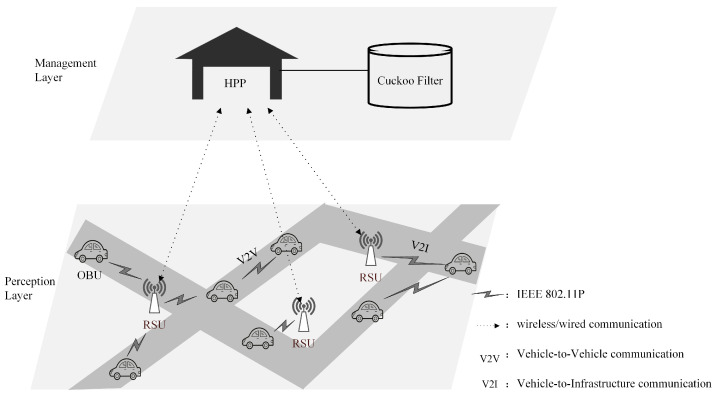
System model.

Figure 1 shows the system model based on the CF, where the OBU registers with the HPP by in-person registration, so the link between the OBU and the HPP is not drawn in the figure. The system model consists of a management layer and a perception layer. The Dedicated Short Range Communication (DSRC) protocol is used to realize the wireless and wired communication between the two layers. The management layer contains the HPP. The perception layer includes OBUs and RSUs. The details of the entities are presented below:

- HPP

The HPP is a trusted third party and is not subject to any threats [17]. It is responsible for the generation of system parameters as well as the registration of entities in VANETs and generates the initial registration information for them. The HPP uses the CF as the internal storage data structure, maintains the CF service, and realizes the internal HomoNym update, query, and HomoNym state change services. A secure channel based on symmetric encryption is implemented between the HPP and RSUs.

- RSU

The RSU is semi-trusted. The communication distance between the RSU and vehicles is at least twice the communication distance between vehicles to ensure that when the RSU receives a message, all vehicles that receive the message are within the jurisdiction of the RSU [18,19]. The RSU has greater computing power than OBUs, which can check the messages’ validity received from vehicles.

- OBU

OBU is a device which is installed in each vehicle. OBU can publish beacons periodically and is used to connect other vehicles. Each OBU has a TPD, which is used to save secure information. Each vehicle carries a root certificate and a pair of manufacturer public and private key pairs (such as $PK_{A}$ and $SK_{A}$ for OBU${}_{A}$) when it leaves the manufacturer.

Based on the IEEE 802.11p communication protocol, VANETs implement real-time communication between vehicles and other vehicles and between vehicles and infrastructure, namely Vehicle-to-Vehicle (V2V) communication and Vehicle-to-Infrastructure (V2I) communication. In addition, as a special type of mobile ad hoc network, VANETs use vehicles as nodes and wireless and wired communication as their communication technology. Its particularity is mainly reflected in the following aspects: (1) high mobility of nodes, (2) regularity of node movement, (3) privacy of node information, (4) frequent exchange of information, (5) differences in traffic scenes, (6) relatively sufficient resources in node, and (7) unstable wireless communication channel  [20,21,22,23].

### 3.2. Cuckoo Filter

The number of HomoNyms in VANETs is constantly growing. Using filters to store HomoNyms in TriNymAuth can effectively save the storage space of HomoNyms in memory, avoid frequent disk reads and writes, and improve the management efficiency of HomoNyms. Using filters can store smaller fingerprints than using a traditional hash table to store HomoNyms. A popular filter data structure is the BF, created in 1970 by Burton Howard Bloom, where each raw data object is mapped to a number of *k* bits in a bit vector, where *k* is the number of independent hash functions. The CF is a compact variant of the Cuckoo hash table; it stores only fingerprints (using the hash function derived from inserting each item as a string) rather than a key-value pair [23]. The fingerprint is calculated by the hash function, usually within 4 bytes, and as long as the selected uniform hash function and the right length of the fingerprint are selected, the hash collision probability can be minimized.The BF does not support entry deletion. The CF not only supports adding and deleting items dynamically but also has greater search performance and better space efficiency. To query whether an item *x* is in a set, simply search the hash table for *x*’s fingerprint and return true if the same fingerprint is found.

The CF can only use two hash functions to calculate candidate indexes, and because these two candidate indexes can be obtained by XOR operation, they are not completely independent, and the length of the filter is limited. Lailong Luo [24] and others believed that the reason for this phenomenon was that the CF had a strong dependence on the length of the filter when calculating the index of the cell or candidate bucket used to store elements. No matter how data sets changed, their capacity must be predefined and kept unchanged. Therefore, they proposed an Index-Independent Cuckoo Filter (I2CF), which decoupled the dependence between bucket index and filter length and realized bucket-level elastic capacity. Further, they organized I2CF into a dynamic list, thus obtaining filter-level elastic capacity. This is the Consistent Cuckoo Filter (CCF) used in this paper.

Algorithms 1–3 present the insert, query, and delete algorithms of the CCF, respectively, where *x* represents inserted data, *f* represents a fingerprint, last represents the last bucket that was replaced, and Max limits the maximum number of iterations. Pagh et al. [25] demonstrated that Max must be set to $\left\lceil 6log_{1+\delta /2}|T|\right\rceil$ in order for the expected time of all operations to be constant, where δ is a small number and |T|≥(2+δ)n. I2CF uses *k* mutually independent hash functions, $h_{i}\left(f\right)\left(1\leq i\leq k\right)$, to compute candidate buckets for fingerprint storage.
**Algorithm 1** Insert(*x*)**Input.**x,last,Max,k **Output.** true/false  1: $f\leftarrow h_{0}\left(x\right)$  2: last←∅  3: **for**
i←0 to Max **do**  4:     calculate hash values $h_{1}\left(f\right),h_{2}\left(f\right),\ldots ,h_{k}\left(f\right)$  5:     acquiring buckets $B_{1},B_{2},\ldots ,B_{k}$ corresponding to hash values from a hash ring  6:     **if** $\exists B_{i}\left(1\leq i\leq k\right)$ contains an empty entry *e* **then**  7:         e←f  8:         **return** true  9:     **end if** 10:     select a fingerprint *e* from any bucket $B_{i}\left(1\leq i\leq k,B_{i}\neq last\right)$ 11:     f↔e 12:     $last↔B_{i}$ 13: **end for** 14: **return** false

**Algorithm 2** Query(*x*)**Input.**x,k **Output.** true/falsev 1: $f\leftarrow h_{0}\left(x\right)$ 2: calculate hash values $h_{1}\left(f\right),h_{2}\left(f\right),\ldots ,h_{k}\left(f\right)$ 3: acquiring buckets $B_{1},B_{2},\ldots ,B_{k}$ corresponding to hash values from a hash ring 4: **if**
$\exists B_{i}\left(1\leq i\leq k\right)$ and $f\in B_{i}$ **then** 5:     **return** true 6: **end if** 7: **return** false

**Algorithm 3** Delete (*x*)**Input.**x,k **Output.** true/false 1: $f\leftarrow h_{0}\left(x\right)$ 2: calculate hash values $h_{1}\left(f\right),h_{2}\left(f\right),\ldots ,h_{k}\left(f\right)$ 3: obtain the bucket $B_{1},B_{2},\ldots ,B_{k}$ 4: **if**
$\exists B_{i}\left(1\leq i\leq k\right)$ and $f\in B_{i}$ **then**. 5:     remove *f* form $B_{i}$ 6:     **return** true 7: **end if** 8: **return** false

### 3.3. Paillier Homomorphic Encryption

Based on the Paillier homomorphic encryption, this paper realizes the generation of the public and private key pair of the HPP, the initial HomoNym generation, the HomoNym update, and the recovery of the real identity of the revoked vehicle. The Paillier homomorphic encryption consists of three steps: (1) PKGA() (Paillier Key Generation Algorithm), (2) Paillier homomorphic encryption Paillier_Enc_PK${}_{P}\left\{msg\right\}$, and (3) Paillier homomorphic decryption Paillier_Dec_SK${}_{P}\left\{C\right\}$.

- PKGA()
  (1) First, choose two large prime numbers, $p_{1}$ and $p_{2}$, for which gcd($\left(p_{1}-1\right)\left(p_{2}-1\right)$) = 1, and calculate $N=p_{1}p_{2}$.
  (2) Second, define L(*x*) = $\frac{\left(x-1\right)}{N}$, select $g\in Z_{N^{2}}^{*}$, where gcd(L($g^{\lambda }$ mod $N^{2}),N)=1$.
  (3) Third, calculate μ = (L($g^{\lambda }$ mod $N^{2}{))}^{-1}$ mod *N*, and λ = LCM$\left(p_{1}-1,p_{2}-1\right)$, where LCM represents the least common multiple.
  (4) Finally, the public key is (N,g), and the private key is (λ,μ).
- Paillier_Enc_PK${}_{P}\left\{msg\right\}$
  The Paillier homomorphic encryption Paillier_Enc_PK${}_{P}\left\{msg\right\}$ is represented in Equation (1). For any plaintext message $msg\in Z_{N}$, a random number $r\in Z_{N}^{*}$ is chosen to calculate the ciphertext *C*.
  (1) $$C=E\left(msg,r\right)=g^{msg}r^{N}\;mod\;N^{2}$$
- Paillier_Dec_SK${}_{P}\left\{C\right\}$
  The Paillier homomorphic decryption Paillier_Dec_SK${}_{P}\left\{C\right\}$ is represented in Equation (2). For the ciphertext $C\in Z_{N^{2}}^{*}$, the plaintext message msg is computed as follows:
  (2) $$msg=D\left(C,\lambda \right)=\frac{L(C^{\lambda }\;mod\;N^{2})}{g^{\lambda }\;mod\;N^{2}}\;mod\;N=L\left(C^{\lambda }\;mod\;N^{2}\right)\cdot \mu \;mod\;N$$

The Paillier homomorphic encryption conforms to the property of additive homomorphism, for any plaintext $msg_{1},msg_{2},\in Z_{N}$, and any $r_{1},r_{2}\in Z_{N}^{*}$, corresponding to the ciphertext $C_{1}=E\left[msg_{1},r_{1}\right],C_{2}=E\left[msg_{2},C_{2}\right]$, satisfying Equation (3).

(3) $$C_{1}\cdot C_{2}=E\left[msg_{1},r_{1}\right]\cdot E\left[msg_{2},r_{2}\right]=g^{msg_{1}+msg_{2}}\cdot {\left(r_{1}\cdot r_{2}\right)}^{N}\;mod\;N^{2}$$

Equation (4) is used to decrypt the ciphertext.

(4) $$D\left[C_{1}\cdot C_{2}\right]=D\left[E\left[msg_{1},r_{1}\right]\cdot E\left[msg_{2},r_{2}\right]\;mod\;N^{2}\right]=msg_{1}+msg_{2}\;mod\;N$$

That is, we obtain $C_{1}\cdot C_{2}=msg_{1}+msg_{2}$. The multiplication of ciphertexts is equal to the addition of plaintexts.

### 3.4. Elliptic Curve Digital Signature Algorithm

In this paper, the vehicle generates LocNyms and local private keys based on the ECDSA [3] Key Generation Algorithm (EKGA()); EKGA() is shown in Algorithm  4. The vehicle realizes identity authentication based on the ECDSA Algorithm ECDSA_Sign_SK${}_{X}\left\{msg\right\}$ to avoid impersonation attacks. ECDSA_Sign_SK${}_{X}\left\{msg\right\}$ is shown in Algorithm 5. The ECDSA Verification Algorithm ECDSA_Verify_PK${}_{X}\left\{msg\right\}$ is shown in Algorithm 6.

Firstly, the global parameters used in Algorithms 4–6 are defined; *q* is a random prime number, an elliptic curve is defined by equation $y^{2}=x^{3}+ax+b$, $a,b\in Z_{q}$; *P* is the base point satisfying the elliptic curve equality, denoted by $P=(x_{g},y_{g})$; *n* is the order of point *P*; that is, *n* is the smallest positive integer satisfying nP=0.
**Algorithm 4** EKGA()**Input.**P,n,q,a,b, **Output.**Q,d 1: select a random integer d∈[1,n−1] 2: calculate Q=dP, obtain a solution point *Q* on the curve $E_{q}\left(a,b\right)$ 3: return vehicle’s local pseudonym is *Q*, local private key is *d*

**Algorithm 5** ECDSA_Sign_SK${}_{X}\left\{msg\right\}$**Input.**n,P,msg,d, **Output.**(r,s) 1: select a random integer or a pseudo-random integer k∈[1,n−1] 2: calculate the solution point of the curve P=(x,y)=kP, and r=xmodn. If r=0, then skip to step 1 3: calculate $t=k^{-1}\;mod\;n$ 4: calculate e=H(msg), where *H* is the hash function SHA-256, which produces a 256-bit hash value 5: calculate $s=k^{-1}\left(e+dr\right)\;mod\;n$. If s=0, then skip to step 1 6: return the signature of msg is (r,s)

**Algorithm 6** ECDSA_Verify_PK${}_{X}\left\{msg\right\}$**Input.**(r,s),n,msg,P,Q **Output.** accept/reject (r,s) 1: check whether *r* and *s* are integers between 1 and n−1 2: calculate a 256-bit Hash value e=H(msg) using hash function SHA-256 3: calculate $w=s^{-1}\;mod\;n$ 4: calculate $u_{1}=ew$ and $u_{2}=rw$ 5: calculate solution point $X=\left(x_{1},y_{1}\right)=u_{1}P+u_{2}Q$ 6: if X=0, reject (r,s), otherwise compute $v=x_{1}\;mod\;n$ 7: if and only if v=r, accept (r,s)


## 4. Pseudonym Management Scheme Based on Paillier Homomorphic Encryption and the CF

In this paper, it is agreed that TriNymAuth satisfies the following assumptions: (1) synchronize the clocks of all entities in VANETs, (2) the HPP is a fully trusted third party that will not be attacked, and (3) RSUs are honest but curious roadside units. TriNymAuth uses the symmetric encryption and public key encryption algorithm used in the WAVE standard protocol, IEEE Std 1609.2-2016 [26]. The symmetric encryption algorithm adopts the advanced encryption standard of a 128-bit key in CCM mode, that is, Advanced Encryption Standard-Counterwith Cipherlockchaining Message (AES-CCM) [27], and the asymmetric encryption algorithm is a P-256 Elliptic Curve Integrate Encrypt Scheme (ECIES) [28]. Moreover, in V2V authentication, in order to prevent impersonation attacks, the vehicle uses the ECDSA algorithm to sign and complete vehicle identity authentication.

**Figure 2:**
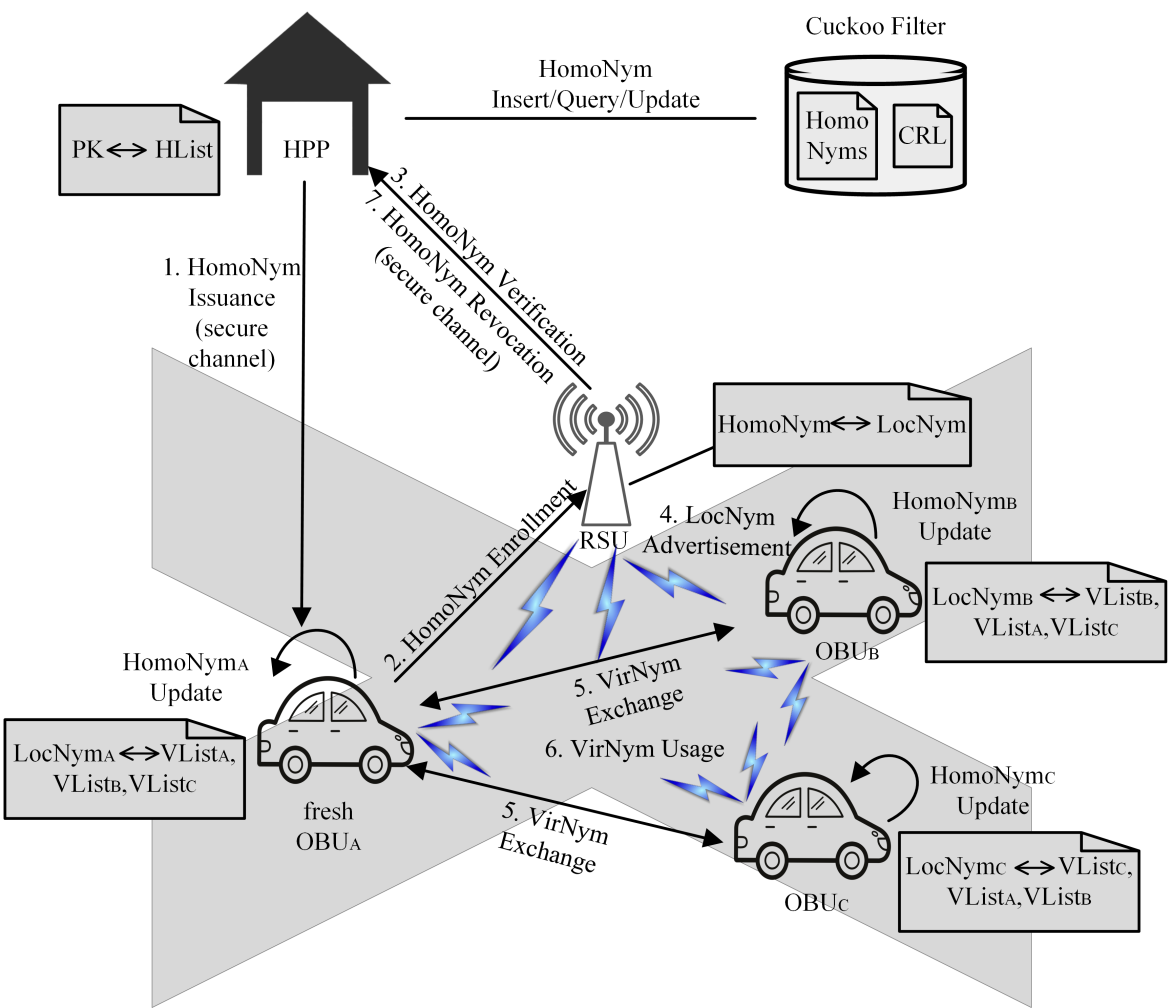
Pseudonym life cycle.

The pseudonym life cycle of TriNymAuth is shown in Figure 2, including the following stages:

- HomoNym issuance. The HPP issues the HomoNym of the vehicle and sends it to the vehicle through a secure channel. After that, the vehicle and the HPP update the pseudonym synchronously, and the HPP calls the insert algorithm of the CF to save the HomoNym in the filter.
- Two-stage HomoNym enrollment. The two-stage HomoNym enrollment consists of: HomoNym verification and LocNym advertisement. Firstly, after the fresh vehicle enters the RSU area, it applies for HomoNym enrollment at the RSU. The RSU forwards the HomoNym to the HPP, and the HPP queries the CF to verify the validity of the HomoNym. After that, the RSU broadcasts the LList in the jurisdiction.
- HomoNym revocation. When there is a malicious vehicle, the vehicle sends the reporting message to the RSU, and the RSU verifies and forwards the HomoNym of the reported vehicle to the HPP, and the HPP performs a revocation operation on the malicious vehicle. In addition, the CF update service is used to update the HomoNym status.
- VirNym exchange. Before V2V communication, vehicles generated VList and exchanged it.
- VirNym usage. For V2V communication, vehicles use VirNym to achieve vehicle identity authentication inside VANETs.

Moreover, the main notations used in TriNymAuth and their descriptions are given in Table 1.

### 4.1. System Initialization

In this phase, the HPP generates basic system parameters and issues public and private keys for the RSU through secure channel.

- HPP selects the cryptographic hash function *h*.
- HPP constructs a multiplicative cyclic group *G* of prime order *l* and generates a random public prime *z* and its public generator ϵ. The HPP constructs Paillier homomorphic encryption Paillier_Enc_PK${}_{P}\left\{msg\right\}$.
- Finally, the HPP publishes the public system parameters, params = {G,h,l,z,ϵ}.
- HPP Key generation. By using PKGA(), the HPP generates its own public key $PK_{P}=\left(N,g\right)$ and private key $SK_{P}=\left(\lambda ,u\right)$.
- RSU Key generation. The HPP chooses a random number $SK_{R}\in {}_{R}Z_{q}$ as the private key of the RSU and computes $PK_{R}=h^{SK_{R}}\in G$ as the public key of the RSU. Based on the secure channel between the HPP and the RSU, the HPP encrypts $PK_{R}$ and $SK_{R}$ with the symmetric key $sym_{HR}$ between them and transmits the ciphertext to the RSU. After receiving it, the RSU decrypts $PK_{R}$ and $SK_{R}$ using $sym_{HR}$.

### 4.2. Homomorphic Pseudonym Generation and SELF-Update Protocol Based on Paillier Homomorphic Encryption

In this phase, based on the secure channel between the HPP and OBU${}_{A}$, the HPP uses symmetric key $sym_{HA}$ to issue the first random seed, update cycle, and initial HomoNym to OBU${}_{A}$ and updates the HomoNyms synchronously with OBU${}_{A}$.

- Initial information issuance:
  1. The first random seed $r_{A0}$ of the OBU${}_{A}$.The HPP randomly selects the first random seed $r_{A0}$ to the OBU${}_{A}$, which is used to update the HomoNym simultaneously between the OBU${}_{A}$ and the HPP.
  2. Update cycle. The HPP specifies the update cycle $T_{U}$[2] for HomoNyms. When the OBU${}_{A}$ receives the initial HomoNym issued by the HPP, it will self-update HomoNym strictly according to the update cycle $T_{U}$ specified by the HPP.
  3. Initial HomoNym. The HPP employs Paillier homomorphic encryption Paillier_E
    nc_PK${}_{P}\left\{msg\right\}$ to generate the OBU${}_{A}$’s initial HomoNym, along with its own public key $PK_{P}$ and randomized seed $r_{A0}$. The calculation formula of the initial HomoNym is represented in Equation (5):
    (5) $$\begin{array}{cc} HomoNym_{A0} & =\;E(ID_{A},r_{A0})\\ \; & =\;{\left[g\;mod\;N^{2}\right]}^{ID_{A}}{\left(r_{A0}\right)}^{N}\;mod\;N^{2} \end{array}$$
  4. Finally, based on the AES-CCM symmetric encryption algorithm, the HPP encrypts $\left\{r_{A0},T_{U},HomoNym_{A0}\right\}$ by using the symmetric key $sym_{HA}$, then sends AES_Enc_ sym${}_{HA}${$r_{A0},T_{U},HomoNym_{A0}$} to the OBU${}_{A}$ through a secure channel. OBU${}_{A}$ executes AES_Dec_sym${}_{HA}${$r_{A0},T_{U},HomoNym_{A0}$} to decrypt and obtain the initial information issued by the HPP and saves in its own TPD.
- HomoNyms self-update
  Based on the update cycle, the vehicles self-update the shared random number and HomoNym synchronously with the HPP, and the calculation formula for the random number and HomoNym is introduced as follows:
  1. Based on the update cycle $T_{U}$, OBU${}_{A}$ self-updates the shared random number synchronously with the HPP according to Equation (6):
    (6) $$\begin{array}{cc} r_{Ai} & =\;E(r_{A(i-1)},sym_{HA})\\ \; & =\;{\left(HomoNym_{A(i-1)}\right)}^{r_{A}\left(i-1\right)}\cdot {\left(sym_{HA}\right)}^{N}\;mod\;N^{2} \end{array}$$
  2. Based on the update cycle $T_{U}$, OBU${}_{A}$ self-updates the $HomoNym_{Ai}$ synchronously with the HPP according to Equation (7):
    (7) $$\begin{array}{cc} HomoNym_{Ai}\; & =\;HomoNym_{A(i-1)}\times E\left(0,r_{Ai}\right)\\ \; & =\;E\left(ID_{A},r_{A(i-1)}\right)\times E\left(0,r_{Ai}\right)\\ \; & =\;E(ID_{A}+0,r^{\prime }) \end{array}$$

After the update is completed, the HPP calls Insert($HomoNym_{Ai}$) (as shown in Algorithm 1) of the CF to insert the $HomoNym_{Ai}$ and the corresponding update cycle $T_{U}$ into the CF.

With the continuous update of HomoNyms, a large number of HomoNyms expire, and deleting expired HomoNyms in real time is conducive to releasing the useful space of the filter reasonably. Because the CF can be deleted, when a HomoNym expires, the expired entry in the filter is backed up first, and then the HPP calls Delete(HomoNym) (as shown in Algorithm 3) of the CF to delete the expired HomoNyms.

### 4.3. Two-Stage Homomorphic Pseudonym Enrollment Protocol Based on the CF

After the vehicle enters VANETs, the initial HomoNym is updated first. The vehicle generates its own LocNym and local private key based on ECDSA Key Generation Algorithm EKGA(), and then the two-stage homomorphic pseudonym enrollment is carried out. The following takes the OBU${}_{A}$ as an example to introduce the whole process.

First, OBU${}_{A}$ inputs $sym_{HA}$ to its TPD, and then the TPD checks whether $sym_{HA}$ matches the symmetric key it has stored. If it does, OBU starts successfully, OBU${}_{A}$ self-updates the initial homomorphic pseudonym $HomoNym_{A0}$, and at the same time OBU${}_{A}$ generates its own $LocNym_{A}$ and ${\hat{SK}}_{A}$ based on the ECDSA Key Generation Algorithm EKGA() (as shown in Algorithm 4). Then, the homomorphic pseudonym verification protocol and the local pseudonym advertisement protocol are executed. In this stage, since OBU${}_{A}$ cannot generate the HomoNyms of other vehicles, it does not need to sign its own HomoNym and LocNym during the homomorphic pseudonym enrollment stage, which reduces the cost of signing and signature verification. In addition, OBU${}_{A}$ updates $HomoNym_{A}$ and $LocNym_{A}$ periodically, and after each update, OBU${}_{A}$ needs to re-enroll.

#### 4.3.1. Homomorphic Pseudonym Verification Protocol Based on the CF

When a fresh vehicle enters an RSU’s jurisdiction, or when the vehicle’s HomoNym and LocNym are updated, the vehicle applies for enrollment using its own HomoNym. The vehicle sends the encrypted HomoNym and LocNym to the RSU. The RSU decrypts it and forwards HomoNym to the HPP, which verifies the validity of the HomoNym through querying the CF and returns the verification result to the RSU. The results are not “expired” or “revoked” if they are valid. The RSU stores the vehicle’s LocNym in LList as well as the table of correspondence between LocNym and HomoNym locally. The following takes the OBU${}_{A}$ as an example to introduce the whole process:

1. After entering the jurisdiction of the RSU or updating $HomoNym_{A}$ and $LocNym_{A}$, based on the ECIES public key encryption algorithm, OBU${}_{A}$ uses the $PK_{R}$ of the RSU to encrypt $HomoNym_{A}$ and $LocNym_{A}$ and sends ECIES_Enc_PK${}_{R}\left\{HomoNym_{A},LocNym_{A}\right\}$ to the RSU. After receiving it, the RSU decrypts ECIES_Dec_SK${}_{R}\left\{HomoNym_{A},LocNym_{A}\right\}$ to obtain $HomoNym_{A}$ and $LocNym_{A}$.
2. Based on the AES-CCM symmetric encryption algorithm, the RSU encrypts its $HomoNym_{A}$ with the symmetric key $sym_{HR}$ and sends the encrypted AES_Enc_sym${}_{HR}\left\{HomoNym_{A}\right\}$ to the HPP. The HPP uses $sym_{HR}$ to decrypt AES_Dec_sym${}_{HR}\left\{HomoNym_{A}\right\}$ to obtain $HomoNym_{A}$. Then the HPP calls the CF’s Query($HomoNym_{A}$) (as shown in Algorithm 2) to query $HomoNym_{A}$. If found, it proves that the $HomoNym_{A}$ is valid, not “expired” or “revoked”. The HPP returns the verification result to the RSU. The RSU keeps the LocNyma of OBU${}_{A}$ in LList and the table of correspondence between $LocNym_{A}$ and $HomoNym_{A}$ locally. If the $HomoNym_{A}$ cannot be found or is expired or revoked, it will be discarded.

**Figure 3:**
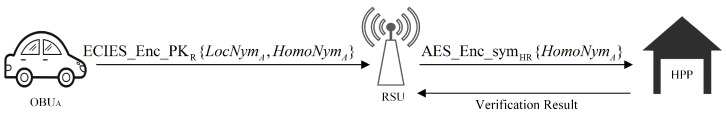
The messages transmitted between entities during the HomoNym enrollment and verification phase.

Figure 3 shows the message transmitted by the VANETs entities during the homomorphic pseudonym enrollment phase, which is steps 2 and 3 in Figure 2. Due to the fact that vehicles cannot generate HomoNyms for other vehicles, OBU${}_{A}$ does not need to sign its $HomoNym_{A}$ and $LocNym_{A}$, but instead must only transmit ECIES_Enc_PK${}_{R}\left\{HomoNym_{A},LocNym_{A}\right\}$ and to the RSU. After the RSU decrypts and obtains $HomoNym_{A}$ and $LocNym_{A}$, $HomoNym_{A}$ will be encrypted to AES_Enc_sym${}_{HR}\left\{HomoNym_{A}\right\}$ and transmitted to the HPP, which reduces the signature overhead of OBU${}_{A}$ and the verification cost of the HPP in this process.

#### 4.3.2. Local Pseudonym Advertisement Protocol

Based on the LocNyms sent by the vehicles during the HomoNym enrollment phase, the RSU integrates LocNyms and generates a LocNym hash list of vehicles, which is broadcast to the vehicles in the jurisdiction. The following takes the OBU${}_{A}$ as an example to introduce the whole process.

Based on the LocNyms sent by the vehicles during the HomoNym enrollment phase, the RSU integrates the LocNyms sent by the vehicles during the HomoNym enrollment phase and generates a LocNym hash list LList of all vehicles within the jurisdiction before broadcasting LList. In addition, when a fresh vehicle enters an RSU’s jurisdiction, or when the vehicle’s HomoNym and LocNym are updated, the RSU updates the LList and broadcasts it. After receiving it, vehicles update the LList stored locally in a time-priority queue manner.

### 4.4. Homomorphic Pseudonym Revocation Protocol Based on Paillier Homomorphic Encryption and the CF

This section illustrates the process of revoking any vehicle that broadcasts false information. Since vehicles communicate with each other using VirNyms, when there is a vehicle broadcasting false information, other vehicles will report the LocNym information corresponding to the VirNym to the RSU, and the RSU will look up the corresponding HomoNym of LocNym in the relation table of HomoNym and LocNym stored locally. Then, the RSU will report the HomoNym to the HPP. The HPP calls the HomoNym state management service of the CF to update the pseudonym state of the malicious vehicle in the revocation period to “revoked”. Finally, the HPP uses its own private key $SK_{P}$ to calculate the vehicle’s real identity based on Paillier homomorphic decryption and carries out the corresponding punishment. The following takes the OBU${}_{A}$ and OBU${}_{B}$ as examples to introduce the whole process:

1. When OBU${}_{B}$ discovers illegal behavior by OBU${}_{A}$, OBU${}_{B}$ uses the $PK_{R}$ of the RSU to encrypt the evidence of violation and the $LocNym_{A}$, $LocNym_{B}$ of OBU${}_{A}$ and OBU${}_{B}$ and sends the ECIES_Enc_PK${}_{R}\left\{evidence,LocNym_{A},LocNym_{B}\right\}$ to the RSU.
2. The RSU decrypts ECIES_Dec_SK${}_{R}\left\{evidence,LocNym_{A},LocNym_{B}\right\}$ and obtains the report message, then looks up $LocNym_{A}$ and $LocNym_{B}$ in the local LList. If the LocNyms are discovered, the identities of OBU${}_{A}$ and OBU${}_{B}$ are established.
3. The RSU finds the $HomoNym_{A}$ of the OBU${}_{A}$ corresponding to $LocNym_{A}$ in the relation table of HomoNym and LocNym stored locally. The RSU encrypts $HomoNym_{A}$ with the symmetric key $sym_{HR}$ and sends AES_Enc_sym${}_{HR}\left\{HomoNym_{A}\right\}$ to the HPP, which decrypts AES_Dec_sym${}_{HR}\left\{HomoNym_{A}\right\}$ and obtains $HomoNym_{A}$ using the AES-CCM symmetric encryption algorithm.
4. According to the $HomoNym_{A}$ of the reported OBU${}_{A}$, the HPP calculates its true identity $ID_{A}$ and all HomoNyms in the revocation period *t* and calls the CF’s pseudonym state change service to set the status of all HomoNyms in the revocation period to “revoked”.
5. According to the additive property of homomorphic encryption, the HPP can obtain the real identity $ID_{A}$ of OBU${}_{A}$ by using Paillier homomorphic decryption Paillier_Dec
  _SK${}_{P}\left\{C\right\}$, which is represented in Equation (8).
  (8) $$\begin{array}{c} D\left(HomoNym_{Ai}\right)=D\left(E\left(ID_{A}+0,r_{Ai}\right)\right) \end{array}$$

### 4.5. V2V Authentication Protocol Based on Virtual Pseudonyms Exchange and Usage

#### 4.5.1. V2V Authentication Protocol Based on Virtual Pseudonyms Exchange

Before communication, the vehicle randomly selects a set of 20-byte random numbers as its own VList based on the random number generator and then exchanges the VList for subsequent mutual identity authentication. Because VirNym is a random number, it effectively prevents impersonation attacks. In V2V communication, the vehicle uses its own local private key to sign. After receiving the signature, other vehicles verify the signature using the vehicle’s LocNym to achieve identity authentication. The following takes OBU${}_{A}$ and OBU${}_{B}$ as examples to introduce the whole process of VirNyms exchange:

1. Before the first communication between OBU${}_{A}$ and OBU${}_{B}$, based on the ECDSA digital signature algorithm, by using OBU${}_{A}$’s local private key ${\hat{SK}}_{A}$, OBU${}_{A}$ executes ECDSA_Sign_${\hat{SK}}_{A}\left\{VList_{A}\right\}$ (as shown in Algorithm 5), the VirNym hash list $VList_{A}$ of OBU${}_{A}$ is signed as ${\left[VList_{A}\right]}_{{\hat{SK}}_{A}}$, and then the signature ${\left[VList_{A}\right]}_{{\hat{SK}}_{A}}$ is sent to OBU${}_{B}$.
2. After receiving it, OBU${}_{B}$ uses the $LocNym_{A}$ of OBU${}_{A}$ to verify the signature ${\left[VList_{A}\right]}_{{\hat{SK}}_{A}}$, executes ECDSA_Verify_LocNym${}_{A}\left\{VList_{A}\right\}$ (as shown in Algorithm 6) to obtain the VirNym hash list $VList_{A}$ of OBU${}_{A}$, and saves it locally.
3. Similarly, by using OBU${}_{B}$’s local private key ${\hat{SK}}_{A}$, OBU${}_{B}$ executes ECDSA_Sign_${\hat{SK}}_{B}\left\{VList_{B}\right\}$ (as shown in Algorithm 5), the VirNym hash list $VList_{B}$ of OBU${}_{B}$ is signed as ${\left[VList_{B}\right]}_{{\hat{SK}}_{B}}$, and then the signature is sent to OBU${}_{A}$.
4. After receiving it, OBU${}_{A}$ uses the $LocNym_{B}$ of OBU${}_{B}$ to verify the signature ${\left[VList_{B}\right]}_{{\hat{SK}}_{B}}$, executes ECDSA_Verify_LocNym${}_{B}\left\{VList_{B}\right\}$ (as shown in Algorithm 6) to obtain the VirNym hash list $VList_{B}$ of OBU${}_{B}$ and saves it locally.

**Figure 4:**
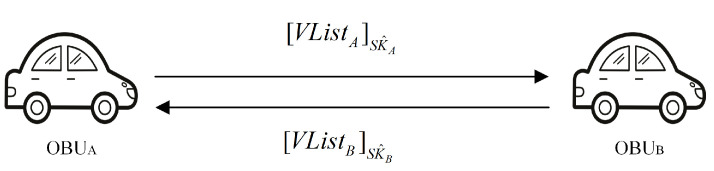
Virtual pseudonyms exchange phase.

Figure 4 illustrates the VirNym hash list VList transmitted between vehicles during the VirNym exchange phase, which is step 5 in Figure 2.

#### 4.5.2. V2V Authentication Protocol Based on Virtual Pseudonyms Usage

When communicating, in order to prevent impersonation attacks, vehicles need to use the ECDSA algorithm to sign VirNyms. The following takes the OBU${}_{A}$ and OBU${}_{B}$ as examples to introduce the whole process:

1. Based on the ECDSA algorithm, OBU${}_{A}$ uses its own local private key ${\hat{SK}}_{A}$ to sign $VirNym_{A}$, and after ECDSA_Sign_${\hat{SK}}_{A}\left\{VirNym_{A}\right\}$ (as shown in Algorithm 5), sends the signature ${\left[VirNym_{A}\right]}_{{\hat{SK}}_{A}}$ and message $msg_{A}$ to OBU${}_{B}$.
2. After receiving them, OBU${}_{B}$ locally queries VList to find LocNym that can verify the signature, and then OBU${}_{B}$ uses the queried $LocNym_{A}$ that corresponds to $VList_{A}$ to verify the signature ${\left[VirNym_{A}\right]}_{{\hat{SK}}_{A}}$, executes ECDSA_Verify_LocNym${}_{A}\left\{VirNym_{A}\right\}$ (as shown in Algorithm 6), and obtains $VirNym_{A}$. Since both $LocNym_{A}$ and $VirNym_{A}$ are from OBU${}_{A}$, it shows that OBU${}_{A}$ did not carry out an impersonation attack.
3. Similarly, based on the ECDSA algorithm, OBU${}_{B}$ uses its own local private key ${\hat{SK}}_{B}$ to sign $VirNym_{B}$, and after ECDSA_Sign_${\hat{SK}}_{B}\left\{VirNym_{B}\right\}$ (as shown in Algorithm 5), sends the signature ${\left[VirNym_{B}\right]}_{{\hat{SK}}_{B}}$ and message $msg_{B}$ to OBU${}_{A}$.
4. After receiving them, OBU${}_{A}$ queries VList locally to find LocNym that can verify the signature, and then OBU${}_{A}$ uses the queried $LocNym_{B}$ that corresponds to $VList_{B}$ to verify the signature ${\left[VirNym_{B}\right]}_{{\hat{SK}}_{B}}$, executes ECDSA_Verify_LocNym${}_{B}\left\{VirNym_{B}\right\}$ (as shown in Algorithm 6), and obtains $VirNym_{B}$. Since both $LocNym_{B}$ and $VirNym_{B}$ are from OBU${}_{B}$, it shows that OBU${}_{B}$ did not carry out an impersonation attack. OBU${}_{A}$ and OBU${}_{B}$ complete the communication.

## 5. Security and Privacy Analysis

The security and privacy analysis of TriNymAuth is performed in this section, and it is demonstrated that TriNymAuth meets almost all of the security and privacy requirements in VANETs. Table 2 compares the TriNymAuth to the related work safety. Comparison results show that TriNymAuth has great superiority.

### 5.1. Security Analysis

In order to achieve secure and efficient vehicle identity authentication, triple pseudonym authentication is used to realize vehicle identity authentication hierarchically and regionally, which decouples internal and external vehicle identities in VANETs. The integrity and non-repudiation of TriNymAuth messages are thus guaranteed, and message tampering attacks are avoided. Meanwhile, the timestamp is included in the message sent by the vehicle, which avoids the replay attack.

**Figure 5:**
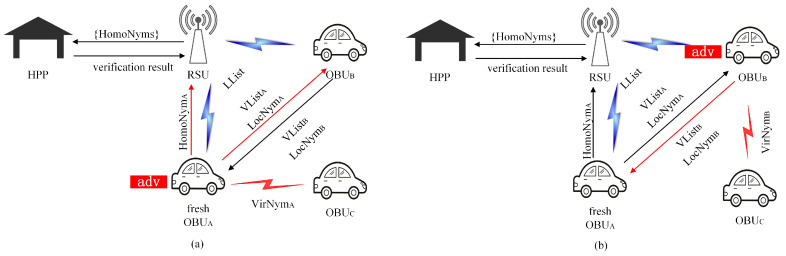
Attack model. The attacker in (**a**) is a fresh OBU${}_{A}$ that joins VANETs. The attacker in (**b**) is the existing OBU${}_{B}$ in VANETs.

In the following, the prevention measures when different attackers carry out impersonation attacks are analyzed according to the attack model shown in Figure 5.

Firstly, when a fresh OBU${}_{A}$ joining the network is the attacker, the impersonation attack of the attacker in different life cycle stages of the pseudonym is analyzed as follows:

1. In the two-stage HomoNym enrollment phase. The adversary OBU${}_{A}$ generates an invalid HomoNym and sends it to the RSU for HomoNym enrollment. The RSU receives it and forwards it to the HPP. The HPP verifies the HomoNym of OBU${}_{A}$. By comparing the HomoNym with the HomoNym stored locally in the HPP, it can be known that the HomoNym used by the OBU${}_{A}$ is invalid, so as to effectively avoid the possible impersonation attack launched by a fresh vehicle in the two-stage HomoNym enrollment phase.
2. In the VirNym exchange phase. The VirNym impersonation attack cannot be carried out in this phase because the OBU${}_{A}$ is a fresh vehicle and cannot obtain the VirNym of other vehicles. In addition, TriNymAuth can effectively avoid the impersonation attack launched by a fresh vehicle in the VirNym exchange phase because the vehicle generates its own local private key based on the EKGA () algorithm, which cannot be obtained by other vehicles through monitoring.
3. In the VirNym usage phase. OBU${}_{A}$ obtains a series of VirNyms of OBU${}_{B}$ in the VirNym exchange phase. If OBU${}_{A}$ wants to pretend to be OBU${}_{B}$, in the VirNym usage phase, OBU${}_{A}$ signs $VirNym_{B}$ using its own local private key ${\hat{SK}}_{A}$ and sends the signature and message to OBU${}_{C}$. Following receipt of the signature, OBU${}_{C}$ searches VList locally for LocNym that can verify the signature, and then OBU${}_{C}$ uses the queried LocNymA to verify the signature and obtain $VirNym_{B}$. OBU${}_{A}$’s impersonation attack failed because OBU${}_{C}$ discovered that $VirNym_{B}$ was not from OBU${}_{A}$. So, TriNymAuth can effectively avoid the possible impersonation attack launched by a fresh vehicle joining VANETs during the VirNym usage phase.

Furthermore, when the OBU${}_{B}$ is the attacker, the impersonation attack is examined at various stages of the pseudonym’s life cycle.

1. In the VirNym exchange phase. The adversary OBU${}_{B}$ sends the $VList_{C}$ of OBU${}_{C}$ to OBU${}_{A}$ as its own VirNym hash list. However, the adversary OBU${}_{B}$ cannot obtain the local private key ${\hat{SK}}_{C}$ of OBU${}_{C}$ and can only sign the $VList_{C}$ using its own local private key ${\hat{SK}}_{B}$, so the impersonation of OBU${}_{C}$ by the adversary OBU${}_{B}$ cannot be successfully implemented.
2. In the VirNym usage phase. The adversary OBU${}_{B}$ obtains the $VList_{C}$ of OBU${}_{C}$ in the VirNym exchange phase. In the VirNym usage phase, OBU${}_{B}$ signs $VirNym_{C}$ using its own local private key ${\hat{SK}}_{B}$ and sends the signature and message to OBU${}_{A}$. After receiving them, OBU${}_{A}$ queries VList locally to find LocNym that can verify the signature, and then OBU${}_{A}$ uses the queried LocNymB to verify the signature and obtain $VirNym_{C}$. OBUB’s impersonation attack failed because OBU${}_{A}$ discovered that $VirNym_{C}$ is not from OBU${}_{B}$. Therefore, TriNymAuth can effectively avoid the possible impersonation attacks launched by vehicles in the VirNym usage phase.

### 5.2. Privacy Analysis

TriNymAuth’s performance in achieving vehicle identity privacy protection, unlinkability, and traceability is analyzed below.

- Identity privacy protection

To begin with, only the HPP is aware of the connection between HomoNym and real identity in terms of identity privacy protection. Based on Paillier homomorphic encryption, the HPP can use its private key to decrypt the HomoNym to obtain the vehicle’s real identity. Since vehicles use VirNyms for communication, HomoNyms are not involved in the communication process. When the adversary is a vehicle, it cannot obtain the vehicle’s HomoNyms through monitoring, so the vehicle’s real identity cannot be further obtained. Therefore, TriNymAuth ensures the vehicle’s identity privacy protection.

- Unlinkability

When the adversary is a vehicle, the link between HomoNyms cannot be implemented because the adversary cannot obtain the HomoNyms of other vehicles. The adversary can link a finite number of VirNyms and LocNyms of other vehicles, and when it comes to the update cycle $T_{U}$ or the vehicle drives across the RSU regions, the VList and LocNym is updated. So, LocNym and VirNym achieve conditional unlinkability. Moreover, the adversary cannot associate the LocNym or VirNym of other vehicles with their HomNym and real identity ID.

- Traceability

Vehicle OBU${}_{A}$ uses $VirNym_{A}$ for V2V communication, and when the adversary is OBU${}_{A}$, the vehicle can obtain the $LocNym_{A}$ of the OBU${}_{A}$ by querying the locally stored VList. Because the RSU saves the correspondence between LocNyms and HomoNyms of the OBU${}_{A}$, when the OBU${}_{A}$ broadcasts false information, the RSU can obtain the $HomoNym_{A}$ of OBU${}_{A}$ by its $LocNym_{A}$ and will forward it to the HPP. The HPP decrypts $HomoNym_{A}$ using its own private key $SK_{P}$, and reveals the real identity $ID_{A}$ of the OBU${}_{A}$. Therefore, TriNymAuth guarantees the traceability of vehicle identity.

## 6. Performance Analysis

Based on the advantages of the CF, we analyze the enrollment cost, computational cost to verify the signature, communication cost in the VirNym exchange phase and usage phase, and the total transmission delay of our scheme. Then, we compare TriNymAuth’s performance with SPECS [5], b-SPECS+ [7], SPACF [13], and VPPCS [6].

### 6.1. Advantage of the CF

The CF adopted in this paper combines a filter-level filter (CCF${}_{F}$) and a bucket-level filter (CCF${}_{B}$). Through experimental analysis, the time cost of TriNymAuth in query and insert is as follows:

#### 6.1.1. Query Overhead

The time complexity of CCF${}_{F}$’s query is about O(k·s·log(m/s)), while the time complexity of CCF${}_{B}$’s query is about O(k·log(m), the former having one more constant factor *s* than the latter. Among them, *m* represents the buckets’ total number in the filter, *s* is the number of I2CF, and *k* is the number of hash functions. Table 3 compares the query overhead (single message) of CCF${}_{F}$ and CCF${}_{B}$.

#### 6.1.2. Insert Overhead

Table 4 compares the average insertion time overhead of CCF${}_{F}$ and CCF${}_{B}$. Since a large number of relocations occur during the insertion of CCF${}_{B}$, the insertion time cost of CCF${}_{B}$ is large This is because a lot of relocations occur during insertion and may be accompanied by the phenomenon of “data migration” [29]. Data migration occurs when the distance between two buckets on the hash ring is so close that most of the data can only be allocated to one bucket. There are enough empty buckets on the hash ring but the load factor is still too high, which leads to the sharp increase in relocation times. Set max to the upper limit of relocation times, *m* is the buckets’ number, and the insertion time complexity of CCF${}_{B}$ is O(max·logm). The insertion time complexity of CCF${}_{F}$ is O(max·log(m/s)), so CCF${}_{F}$’s insertion time is smaller than CCF${}_{B}$, and the smaller *s* is, the less time it takes to insert, but the cost is increased query and delete time because they need to traverse all I2CF Consider extreme s=m, it is not hard to think the filter will retreat into a linked list or query, and the delete time complexity will increase to a linear level.

TriNymAuth uses the CF combining CCF${}_{F}$ and CCF${}_{B}$, and the actual time cost is between them about 10 μs.

#### 6.1.3. Space Overhead

**Figure 6:**
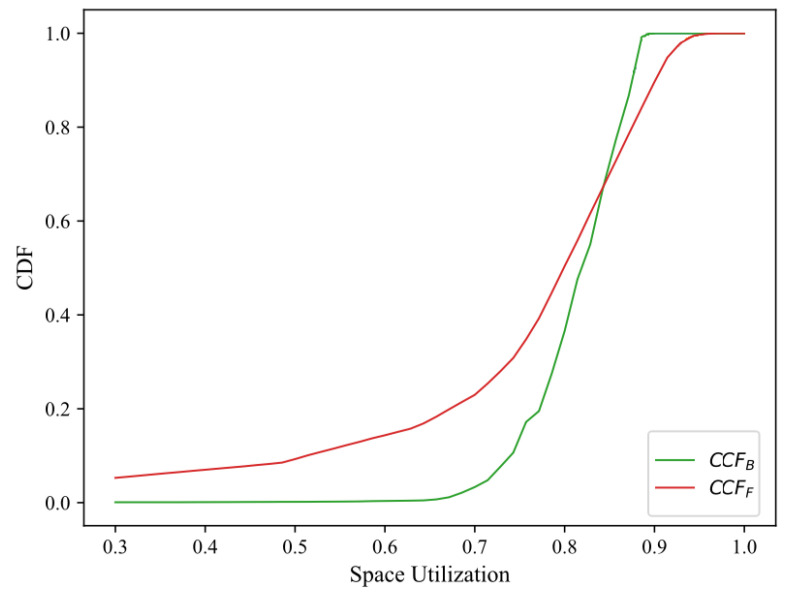
CDF of space utilization of CCF${}_{F}$ and CCF${}_{B}$ under the same workflow.

Figure 6 analyzes the cumulative distribution of space utilization for CCF${}_{F}$ and CCF${}_{B}$. It can be seen that the space utilization of CCF${}_{F}$ and CCF${}_{B}$ exceeds 80% in about 50% and 63% of the cases, respectively. The CF combined with CCF${}_{F}$ and CCF${}_{B}$ has better elasticity and higher space utilization, which makes it more suitable for dynamic data set representation. In VANETs, improving the space utilization of the HomoNym management system can make the system have more free memory and execute more complex tasks at the same time.

#### 6.1.4. False Positive Rate

TriNymAuth randomly selects a byte from the HomoNym of the original data set and performs an XOR operation with another randomly generated byte. TriNymAuth replaces the result with the selected byte in the HomoNym, obtains the test data set, and removes the intersection with the original data set. After inserting the original data set’s HomoNyms into the CF, the query operation is performed on the HomoNyms in the test data set in the filter. When the fingerprint length is 16 bits or 32 bits, the false positive rates of CCF${}_{F}$ and CCF${}_{B}$ are 0.6204 and 0.0014, respectively.

Compared with the fingerprint length of 16 bits, when the fingerprint length is 32 bits, the false positive rate of CCF${}_{F}$ and CCF${}_{B}$ is reduced to about 0.1%, which is significantly improved. Therefore, the fingerprint length should be greater than or equal to 32 bits in practice.

### 6.2. Enrollment Cost Analysis and Comparison

TriNymAuth protocols are performed on a simulator, which is written in C++. We suppose that the jurisdiction area of the RSU is a circle with a radius of 1 km, and the travel range of vehicles through the RSU is 0 to 2 km. The vehicle travels at random speeds ranging from 5 m/s to 40 m/s (20–144 km/h).

In SPECS [5], b-SPECS+ [7], and SPACF [13], the enrollment cost is divided into two parts: (1) First is the initial handshake cost. The initial handshake is performed when a vehicle enters the jurisdiction of a new RSU. Although the initial handshake only needs to be performed once in the whole system, it also incurs a large computational overhead. In this process, the initial handshake cost of a vehicle is made up of one ECC encryption and decryption, one signature, and one signature verification [5,7,13]. Thus, the whole phase’s total computation time is as follows:

$$4T_{e\cdot m}+T_{h}+\left(2T_{bp}+2T_{b\cdot pm}+T_{mtp}+T_{h}\right)\approx 18.0213\;\mathrm{ms}$$

where $T_{e\cdot m}$ is the Elliptic Curve Cryptography (ECC)-based scale multiplication operation’s execution time, $T_{h}$ is the one-way hash function operation’s execution time, $T_{bp}$ is the bilinear pairing operation’s execution time, $T_{b\cdot pm}$ is the bilinear pairing-based scale multiplication operation’s execution time, $T_{mtp}$ is the bilinear pairing-related MapToPoint hash operation’s execution time. The initial handshake cost of the RSU is made up of one ECC decryption and one signature verification [5,7,13]. Thus, the whole phase’s total computation time is as follows:

$$2T_{e\cdot m}+\left(2T_{bp}+2T_{b\cdot pm}+T_{mtp}+T_{h}\right)\approx 17.1372\;\mathrm{ms}$$

The initial handshake cost of the TA is made up of two ECC encryption, one ECC decryption, one signature, and one signature verification [5,7,13]. Thus, the whole phase’s total computation time is as follows:

$$6T_{e\cdot m}+T_{h}+\left(2T_{bp}+2T_{b\cdot pm}+T_{mtp}+T_{h}\right)\approx 18.9053\;\mathrm{ms}.$$

(2) Second is the periodic handshake cost. For the shared secret with the RSU, a new secret is generated every time the vehicle moves into the region of another RSU. At this time, the vehicle needs to perform encryption and decryption operations with the TA. In this process, the periodic handshake cost of a vehicle is made up of one ECC encryption and decryption, one signature, and one signature verification [5,7,13]. Thus, the whole phase’s total computation time is as follows:

$$4T_{e\cdot m}+\left(2T_{bp}+2T_{b\cdot pm}+T_{mtp}+T_{h}\right)\approx 18.0212\;\mathrm{ms}$$

The periodic handshake cost of the RSU is made up of one ECC decryption and one signature verification [5,7,13]. Thus, the whole phase’s total computation time is as follows:

$$2T_{e\cdot m}+\left(2T_{bp}+2T_{b\cdot pm}+T_{mtp}+T_{h}\right)\approx 17.1372\;\mathrm{ms}$$

The periodic handshake cost of the HPP is made up of two ECC encryption, one ECC decryption, and one signature [5,7,13]. Thus, the whole phase’s total computation time is as follows:

$$6T_{e\cdot m}+T_{h}\approx 2.6521\;\mathrm{ms}$$

In VPPCS [6], the enrollment cost of a vehicle is made up of one ECC encryption and decryption, one signature, and one signature verification [6]. Thus, the whole phase’s total computation time is as follows:

$$4T_{e\cdot m}+T_{h}+\left(2T_{bp}+2T_{b\cdot pm}+T_{mtp}+T_{h}\right)\approx 18.0213\;\mathrm{ms}$$

The enrollment cost of the RSU is made up of one ECC encryption and decryption, one signature, and one signature verification [6]. Thus, the whole phase’s total computation time is as follows:

$$4T_{e\cdot m}+T_{h}+\left(2T_{bp}+2T_{b\cdot pm}+T_{mtp}+T_{h}\right)\approx 18.0213\;\mathrm{ms}$$

In the HomoNym enrollment phase of TriNymAuth, the enrollment cost can be divided into the following parts. First, based on the ECIES public key encryption algorithm, the vehicle encrypts the HomoNym and LocNym and forwards them to the RSU. The RSU decrypts and obtains HomoNym and LocNym. Secondly, based on the AES-CCM symmetric encryption algorithm, the RSU encrypts the homonym and forwards it to the HPP, which decrypts it and obtains HomoNym. The HPP verifies the validity of HomoNym by querying the CF. Finally, based on the AES-CCM symmetric encryption, the HPP encrypts the verification result and sends it to the RSU. The experiments show that the AES-CCM symmetric encryption algorithm with a 128-bit key length can encrypt and decrypt a 128-byte message in time $T_{AES}^{enc}=0.5855$ ms and $T_{AES}^{dec}=0.5375$ ms, respectively. The enrollment cost of the vehicle is made up of one ECIES encryption in this process. Thus, the whole phase’s total computation time is as follows:

$$2T_{e\cdot m}\approx 0.8840\;\mathrm{ms}$$

The enrollment cost of the RSU is made up of one ECIES decryption, one AES symmetric encryption and decryption. As a result, the total computation time for the entire phase is as follows:

$$2T_{e\cdot m}+T_{AES}^{enc}+T_{AES}^{dec}\approx 2.0070\;\mathrm{ms}$$

The enrollment cost of the HPP is made up of one AES symmetric encryption and decryption and one CF query. Thus, the whole phase’s total computation time is as follows:

$$T_{AES}^{enc}+T_{AES}^{dec}+0.5\times \left(2.934+1.689\right)\times 10^{-3}\approx 1.1253\;\mathrm{ms}$$

The cost of the HomoNym enrollment phase only includes ECIES and AES-CCM symmetric encryption and decryption costs and the CF query costs, which do not involve the overhead of signature and signature verification. It greatly improves the enrollment efficiency of vehicles and reduces the computational cost.

Table 5 analyzes and compares the specific overhead of our scheme, TriNymAuth, and SPECS [5], b-SPECS+ [7], SPACF [13], and VPPCS [6] in the enrollment phase.

### 6.3. Computational Cost Analysis and Comparison

The traditional anonymous identity authentication scheme uses complex mathematical calculations (such as bilinear pairing and so on) to achieve identity authentication, which has a large computational overhead. In this paper, vehicles use ECDSA-based signatures to achieve identity authentication, which greatly reduces the overhead.

SPECS [5] and b-SPECS+ [7] are established on cryptographic operations based on bilinear pairing; however, SPACF [13], VPPCS [6], and TriNymAuth are established on ECC-based cryptographic operations.

In SPECS [5] and b-SPECS+ [7], the computational overhead of single message verification is made up of one one-way hash function operation, one bilinear pairing-related MapToPoint hash function operation, one bilinear pairing-related point addition operation, two bilinear pairing operations, and two bilinear pairing-related scalar multiplication operations. Thus, the whole phase’s total computation time is as follows:

$$T_{h}+T_{mtp}+T_{bp\cdot a}+2T_{bp}+2T_{b\cdot pm}\approx 16.2532\;\mathrm{ms}$$

where $T_{bp\cdot a}$ is the bilinear pairing-based point addition operation’s execution time.

In SPACF [13], the computational overhead of single message verification is made up of one one-way hash function operation, one ECC-related point addition operation, and two ECC-related scalar multiplication operations. Thus, the whole phase’s total computation time is as follows:

$$T_{h}+T_{e.a}+2T_{e.m}\approx 0.8859\;\mathrm{ms}$$

In VPPCS [6], the computational overhead of single message verification is made up of one one-way hash function operations, one-point addition operations, and two ECC-related scalar multiplication operations. Thus, the whole phase’s total computation time is as follows:

$$T_{h}+T_{e.sm}+2T_{e.m}\approx 0.8979\;\mathrm{ms}$$

In our scheme, TriNymAuth, the computational overhead of single message verification is made up of two double one-point multiplications over an elliptic curve. Thus, the whole phase’s total computation time is as follows:

$$2\times (2T_{mul})\approx 1.5600\;\mathrm{ms}$$

where $T_{mul}$ is the one-point multiplication’s execution time.

### 6.4. Communication Cost Analysis and Comparison

This section analyzes the communication cost of TriNymAuth. Since vehicles need to carry out the VirNym exchange phase before communication, the communication cost of TriNymAuth is divided into two parts: (1) the communication cost of the VirNym exchange, and (2) the communication cost of a single beacon. The comparison between TriNymAuth and SPECS [5], b-SPECS+ [7], SPACF [13], and VPPCS [6] is also given when analyzing the communication overhead of a single beacon.

#### 6.4.1. VirNym Exchange Communication Cost

Different from other anonymous authentication schemes, in TriNymAuth, OBU${}_{A}$ needs to exchange VList through the VirNym exchange phase before communication, which is used to realize the identity authentication during the VirNym use phase. TriNymAuth generates random numbers for anti-collision based on SHA-1, and the number of bits of VirNyms needs to be greater than or equal to 20 bytes. In the VirNym exchange stage, the content of OBU${}_{A}$ broadcast to the verifier is $VList_{A}$, and the communication overhead is 20c bytes, where *c* is the number of VirNyms. Based on the security certificate management system (SCMS) in the United States, the pseudonym replaces the simple specification and is stored by encryption. When the vehicle moving distance is more than 2 km and it is mobile for more than five minutes [2], the RSU jurisdiction is replaced. Otherwise, an hour later LocNyms will be replaced. So, the c=12, VirNym exchange stage of the communication overhead is 240 bytes, and the communication overhead is reduced while ensuring the number of VirNyms needed for vehicle communication.

#### 6.4.2. Single Beacon Transfer Communication Cost

In this section, taking OBU${}_{A}$ as an example, TriNymAuth is compared with SPECS [5], b-SPECS+ [7], SPACF [13], and VPPCS [6] regarding communication cost. For convenience, we assume that the scheme environment is the same for all schemes discussed below. Since the vehicles in the scheme all involve messages when communicating, the communication overhead of messages is temporarily not considered in the comparative discussion.

In SPECS [5] and b-SPECS+ [7], the content that OBU${}_{A}$ broadcasts to the verifier is $\left\{ID_{i},M_{i},\sigma_{i}\right\}$, where $ID_{i}$ is the anonymous identity, $M_{i}$ is the message, and $\sigma_{i}$ is the signature. $ID_{i}=\left(ID_{i1},ID_{i2}\right)$ and $ID_{i1},ID_{i2}\in G_{1}$. $\sigma_{i}=SK_{i1}+h\left(M_{i}\right)SK_{i2}$ and $\sigma_{i}\in G_{1}$. Therefore, the communication cost is 128×3=384 bytes.

In SPACF [13], the content that OBU${}_{A}$ broadcasts to the verifier is $\left\{M_{i},T_{i},ID_{i},\sigma_{i}\right\}$, where $M_{i}$ is the message, $T_{i}$ is the timestamp, $ID_{i}$ is the anonymous identity, $ID_{i}=\left(ID_{i1},ID_{i2}\right),ID_{i1}=a\cdot PK_{P}\in G,ID_{i2}=PK⊕h\left(m_{i}\cdot ID_{i1}\right)\in Z_{q}^{*}$, $\sigma_{i}$ is the signature, $\sigma_{i}=r_{i}+m_{i}H_{2}(ID_{i}\left|\right|M_{i}\left|\right|T_{i})\;mod\;q$, and $m_{i}$ is the new secret value. Therefore, the communication overhead is 40×1+20×2+4=84 bytes.

In VPPCS [6], the content that OBU${}_{A}$ broadcasts to the verifier is $\left\{PID_{il}^{1},PID_{il}^{2},\sigma_{m},T,T_{SK_{il}}\right\}$, where $PID_{il}^{1},PID_{il}^{2}$ are the anonymous identities, $\sigma_{m}$ is the signature, and $T,T_{SK_{il}}$ are two timestamps. $PID_{il}^{1}\in G$ and $PID_{il}^{2},\sigma_{m}\in Z_{q}^{*}$. Therefore, the communication cost is 40+20×2+2×4=88 bytes.

In the VirNym usage phase of TriNymAuth, the content that OBU${}_{A}$ broadcasts to the verifier is $\left\{m_{i},{\left[VirNym_{A}\right]}_{{\hat{SK}}_{A}},T_{A}\right\}$, where *m* is the message, ${\left[VirNym_{A}\right]}_{{\hat{SK}}_{A}}$ is the signature, and $T_{A}$ is the timestamp, therefore, the communication overhead is 64+4=68 bytes.

VC represents the verification cost (ms), CC represents the communication cost (bytes), and TTD represents the total transmission delay (ms). The comparison of our scheme TriNymAuth and SPECS [5], b-SPECS+ [7], SPACF [13], and VPPCS [6] in terms of VC, CC and TTD is given in Table 6, where dr stands for data rate in VANETs.

Based on the formula “TTD = $\frac{CC\times 8}{dr}$ + VC”, the TTD of SPECS [5] and b-SPECS+ [7] is expressed as $\frac{384\times 8}{dr}+16.2532$ (ms), the TTD of SPACF [10] is expressed as $\frac{84\times 8}{dr}+0.8859$ (ms), the TTD of VPPCS [6] is expressed as $\frac{88\times 8}{dr}+0.8979$ (ms), and the TTD of TriNymAuth is denoted by $\frac{68\times 8}{dr}+1.5600$ (ms). When the transmission distance is less than 300 m, the transmission rate of 802.11p in the 915 MHz band is lower than 500 kbps [30]. The calculation shows that when the data rate in VANETs dr≤ 500 kbps, TriNymAuth’s total transmission delay is better than SPECS [5] and b-SPECS+ [7], and when dr≤ 180 kbps, TriNymAuth’s total transmission delay cost is the lowest of the five. Therefore, TriNymAuth is suitable for shopping malls and other places with dense traffic.

## 7. Conclusions

This paper proposes a triple pseudonym authentication scheme for VANETs based on the CF and Paillier homomorphic encryption (TriNymAuth). Paillier homomorphic encryption is used to achieve an efficient HomoNym self-update. The HPP can efficiently verify the validity of HomoNyms based on the CF queries. TriNymAuth uses HomoNym, LocNym, and VirNym to realize the triple authentication of vehicle identity, which improves the authentication efficiency while ensuring the privacy and security of vehicle identity. The experimental results show that the CF performs well in terms of insertion and query time, space utilization, and false positive rate. In particular, the CF achieves 10 μs insertion overhead, and the query overhead reaches the level of ns. Moreover, in the case of more than 50%, the space utilization exceeds 80%, and the space utilization of the scheme is high. The false positive rate is as low as 1% when the fingerprint length is 32 bits. Comparing the enrollment, verification, and communication costs of TriNymAuth with those of SPECS [5], b-SPECS+ [7], SPACF [13], and VPPCS [6], it can be seen that TriNymAuth has great advantages in the enrollment cost, and when the data rate in VANETs dr≤ 180 kbps, TriNymAuth has the smallest total delay cost and is suitable for shopping malls and other places with dense traffic.

## Figures and Tables

**Table 1:** Scheme notation and its description.

Notation	Description
$HList_{A}=\left\{HomoNym_{A}\right\}$	A hash list of HomoNyms of OBU${}_{A}$
$VList_{A}=\left\{VirNym_{A}\right\}$	A hash list of VirNyms of OBU${}_{A}$
LList={LocNym}	A hash list of LocNyms of OBU under the jurisdiction of an RSU
$r_{A0}$	The first random seed of OBU${}_{A}$
$sym_{XY}$	The symmetric key between *X* and *Y*
AES_Enc_sym${}_{XY}\left\{msg\right\}$	Using the symmetric key $sym_{XY}$ to encrypt message msg
AES_Dec_sym${}_{XY}\left\{C\right\}$	Using the symmetric key $sym_{XY}$ to decrypt ciphertext *C*
ECIES_Enc_PK${}_{X}\left\{msg\right\}$	Using the public key $PK_{X}$ of *X* to encrypt message msg
ECIES_Dec_SK${}_{X}\left\{C\right\}$	Using the private key $SK_{X}$ of *X* to decrypt ciphertext *C*
Paillier_Enc_PK${}_{X}\left\{msg\right\}$	Using the public key to Paillier homomorphic encrypt message msg
Paillier_Dec_SK${}_{X}\left\{C\right\}$	Using the private key $SK_{X}$ of *X* to Paillier homomorphic decrypt ciphertext *C*
ECDSA_Sign_${\hat{SK}}_{X}$/SK${}_{X}\left\{msg\right\}$	Using the local private key ${\hat{SK}}_{X}$ of OBU${}_{X}$ or the manufacturer private key $SK_{X}$ of OBU${}_{X}$ to sign msg with ECDSA
ECDSA_Verify_LocNym${}_{X}$/PK${}_{X}\left\{msg\right\}$	Using the local public key $LocNym_{X}$ of OBU${}_{X}$ or the manufacturer public key $PK_{X}$ of OBU${}_{X}$ to do ECDSA signature verification on msg

**Table 2:** Security and privacy comparison.

Security	SPECS [5]	b-SPECS+ [7]	SPACF [13]	VPPCS [6]	TriNymAuth
Resistance to impersonation attack	-	✓	-	✓	✓
Identity privacy protection	✓	✓	✓	✓	✓
Traceability	✓	✓	✓	✓	✓
Unlinkability	-	-	✓	✓	✓

**Table 3:** The query overhead of CCF${}_{F}$ and CCF${}_{B}$ (ns).

CF	Query Time (ns)
CCF${}_{F}$	2934
CCF${}_{B}$	1689

**Table 4:** The average insert overhead of CCF${}_{F}$ and CCF${}_{B}$(μs).

CF	Insert Time (μs)
CCF${}_{F}$	2.5
CCF${}_{B}$	11.7

**Table 5:** Enrollment cost analysis and comparison (ms).

Schemes	Phase	Entity	Enrollment Cost (ms)
SPECS [5] b-SPECS+ [7] SPACF [13]	Initial handshake	OBU	18.0213
		RSU	17.1372
		TA	18.9053
	periodic handshake	OBU	18.0212
		RSU	17.1372
		TA	2.6521
VPPCS [6]	periodic enrollment	OBU	18.0213
		RSU	18.0213
TriNymAuth	periodic enrollment	OBU	0.8840
		RSU	2.0070
		HPP	1.1253

**Table 6:** Comparison of verification cost, communication cost, and total transmission delay.

Schemes	VC	CC	TTD (dr = 500 kbps)	TTD (dr = 180 kbps)
SPECS [5], b-SPECS+ [7]	16.2532	384	22.3972	33.3199
SPACF [13]	0.8859	84	2.2299	9.2859
VPPCS [6]	0.8979	88	2.3059	4.8090
TriNymAuth	1.5600	68	2.6480	4.5820

## Data Availability

Not applicable.
